# Dynamics of Virus-Receptor Interactions in Virus Binding, Signaling, and Endocytosis

**DOI:** 10.3390/v7062747

**Published:** 2015-06-02

**Authors:** Steeve Boulant, Megan Stanifer, Pierre-Yves Lozach

**Affiliations:** 1 CellNetworks-Cluster of Excellence and Department of Infectious Diseases, Virology, University Hospital Heidelberg, 69120 Heidelberg, Germany; E-Mail: m.stanifer@dkfz-heidelberg.de (M.S.); 2 Schaller research group at CellNetworks and DKFZ (German cancer research center), 69120 Heidelberg, Germany

**Keywords:** binding, endocytosis, signalization, single particle tracking, uptake, virus receptor

## Abstract

During viral infection the first challenge that viruses have to overcome is gaining access to the intracellular compartment. The infection process starts when the virus contacts the surface of the host cell. A complex series of events ensues, including diffusion at the host cell membrane surface, binding to receptors, signaling, internalization, and delivery of the genetic information. The focus of this review is on the very initial steps of virus entry, from receptor binding to particle uptake into the host cell. We will discuss how viruses find their receptor, move to sub-membranous regions permissive for entry, and how they hijack the receptor-mediated signaling pathway to promote their internalization.

## 1. Introduction

Viruses are strict intracellular parasites and their life cycle is fully reliant on hijacking cellular functions to promote their replication and spread. Virus entry is the very first step of viral infection. Tremendous effort has been made to characterize the cellular receptors and the entry pathways that mediate virus internalization. With the development of high-throughput technologies and haploid genetic screens, a plethora of virus receptors and their associated endocytic pathways have been identified in the last few years [1,2,3,4]. While this growing knowledge offers novel potential therapeutic strategies, very little is understood about the dynamic molecular processes of virus-receptor interaction and virus internalization.

This review will focus on the very early steps of receptor-mediated endocytosis of viruses, from receptors binding to the physical internalization of the viral particle into the host cells. We will discuss the current knowledge of the dynamic process of virus/cell surface and virus/receptor interaction. Additionally, we will detail our current understanding of the molecular signaling strategies that viruses utilize to promote their specific endocytosis and the endocytic/sorting motifs of receptors that are exploited by viruses for infection.

## 2. Virus Entry is a Complex Multistep Process

**Figure 1:**
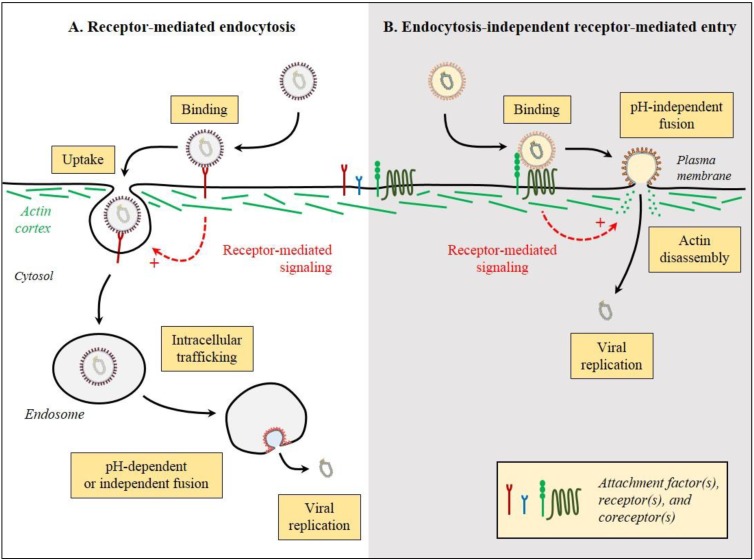
Strategies of virus entry. To gain access to the cytoplasm of host cells, viruses can employ two main strategies, *i.e.*, either (**A**) through endocytosis and escape from endosomal vesicles in a process referred as receptor-mediated endocytosis or (**B**) by direct penetration from the plasma membrane, referred as endocytosis-independent receptor-mediated entry. Enveloped viruses are shown; however non-enveloped viruses have evolved similar strategies. These are just generalizations and there are exemptions from these rules. Black arrows represent the sequence of events and dashed-red arrows the potential induced signaling.

To establish infection and replicate, viruses need to gain access to the intracellular environment. This very first step is strictly dependent on surface exposed cellular receptors to which virus particles bind. Viruses can use two different strategies to enter the host. First, in the classical virus endocytosis model, following binding to one or multiple cellular receptors, virus particles are physically up taken by the endocytic cellular machinery in a process referred to as receptor-mediated endocytosis (Figure 1A). In a second strategy, virus binding to cellular receptors leads to the direct penetration of the virus particles from the plasma membrane, bypassing the endocytic machinery. This process is referred to as endocytosis-independent receptor-mediated entry (Figure 1B). While the receptor-mediated endocytosis model has the merit of being conceptually simple, it is a complex multistep process where viruses are faced with fundamental challenges in order to hijack the host endocytic machinery. First, viruses need to gain access to the cell surface before binding their receptor. This primary attachment is often facilitated by attachment factors that mediate the non-specific binding of virus particles allowing their concentration at the cell surface. These attachments factors are usually small, charged proteins, lipids, or sugar moieties (*i.e.*, heparin sulfate, sialic acid, gangliosides) to which virus particles can bind electrostatically. Following this primary attachment, the virus particles have to interact with the specific virus receptor(s) in order to be internalized. Emerging evidence indicates that cell signaling is strongly activated during viral infection and might facilitate viral uptake and appropriate intracellular targeting [5]. It is often assumed that an active out-in signaling through the receptor molecules triggers the internalization of virus particles by the cellular uptake machinery. Although we have a lot of information on virus/receptor interactions at the molecular and structure level, our understanding of the mechanisms by which virus/receptor interactions induce signaling pathways and how these might actively mediate internalization of the virus/receptor complex is very limited. Conversely, the molecular motifs in a receptor that drive signaling and physical internalization are poorly characterized. Interestingly, several lines of evidence support that all virus/receptor interactions do not always lead to active uptake of the virus particles and that some viruses depend on stochastic uptake by the host cell without relying on active signal induction. Concrete demonstrations that a receptor-mediated signaling results in active virus uptake and productive infection are missing. It is often not clear whether activation of specific cellular signaling pathways drives or results from the endocytic event.

## 3. Virus Movement on the Cell Surface

Live microscopy with high spatio-temporal resolution and single virus particle tracking has enabled researchers to follow viruses from the very first moment they interact with the cells (*i.e.*, landing on the cell surface or transmission of virus particles to uninfected cells via viral synapses) to the moment the virus particles are physically endocytosed by the host cells [6,7,8,9]. Characterization of the very first interaction step between viruses and their attachment factors or receptors have been limited due to the technical challenge of tracking virus particles from the medium to the surface of the cells. However, this step has been extensively studied *in vitro* and follow a classical thermodynamic rule [10,11,12,13,14,15,16,17]. The more attachment factor or receptor molecules are present at the cell surface, and the more affinity a virus has for this receptor, the more efficient the primary interaction will be.

Analysis of the diffusion/mobility of viral particles at the cell surface revealed that virus journey, from cell attachment to endocytosis, is a complex multistep process. Three distinct mobility profiles have been observed for virions following landing on host cells, the type of which most likely depends on the strategy used by the virus to be internalized. The first and simplest mobility profile is random motion/walk or diffusion where particles move without apparent order [18]. The second possible movement is constrained diffusion where the virus particles seem to be restricted to a very precise microdomain of the cellular membrane. In the third mobility profile, particles display directional movement or drifts following a precise track or direction, such as viruses moving in a retrograde flow along filopodia [19]. These different motilities are the consequence of the heterogeneous nature of the plasma membrane, which is organized in microdomains or rafts [20]. Protein and lipid diffusion is restricted and confined by these domains and is intrinsically linked to the underlying cortical actin network [21,22,23,24].

## 4. Virus Binding to Receptor: The Land and Stick Approach *vs.* the Land and Seek Approach

**Figure 2:**
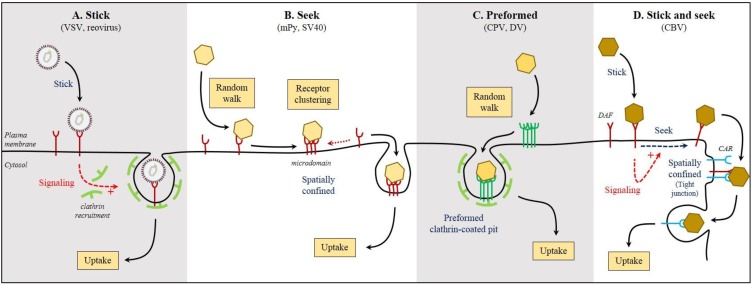
How viruses find their receptor(s). Viruses have evolved different strategies to interact with their receptor(s) at the surface of host cells. (**A**) After landing on the cell surface and binding to receptor(s), some viruses remain stick at a confined location from where they will be endocytosed in a passive or signal-induced manner; (**B**) Others, after binding to their receptor, will diffuse at the cell surface seeking additional receptor molecules. The virus/receptor complex will be then spatially confined in a host plasma membrane microdomain from where it will be endocytosed; (**C**) Some viruses, following binding to their receptors, diffuse at the surface of the host cell seeking for preformed endocytic structures that they hijack to mediate their internalization; (**D**) Viruses can land on the surface of the cells and bind to a primary receptor. This initial binding induces signaling that leads to active redistribution of the virus/primary receptor complex seeking for a secondary receptor that mediates virus uptake. *Abbreviations*: CAR, coxsackievirus and adenovirus receptor; CBV, group B coxsackievirus; CPV, canine parvovirus; DAF, CBV co-receptor; DV, dengue virus; mPy, mouse Polyomavirus; SV40, simian polyomavirus 40; VSV, vesicular stomatitis virus.

The simplest scenario of virus/cell surface attachment, interaction with receptor, and endocytosis would be that the virus particles remain spatially confined immediately following interaction with the cellular surface (Figure 2A). In this case, virus particles have already attached to their receptor and are waiting to be endocytosed, or they have attached to an attachment factor or primary receptor and are waiting for the stochastic or induced recruitment of a secondary receptor. Reovirus and vesicular stomatitis virus (VSV) are examples of virus particles that display confined displacement following cell surface attachment prior to their internalization by the well-characterized clathrin-mediated endocytosis (CME) pathway [7,8,25]. In both cases, it is unclear what happens between cell attachment and physical internalization. Very few studies have successfully addressed this complex step. For reovirus the following model has been suggested: after binding of reovirus to its cell attachment factor sialic acid, the virus is able to bind its co-receptor β1 integrin. This binding would drive the recruitment and clustering of the junctional adhesion molecule A (JAM-A), which would mediate internalization of the virus/receptor complex [13,26,27,28]. Although this model looks very satisfactory, the dynamic recruitment of the various receptors and their clustering has not been monitored. It remains unclear whether the virus/primary receptor complex moves to the secondary receptor(s) or whether the secondary receptors move toward the virus/primary receptor complex. Most of our current models of dynamic virus/receptor interaction and internalization are putative series of events based on biochemical and genetic information but not based on dynamic interaction data.

In other instances, following attachment to the cell surface, viruses display a complex multistep mobility profile. They can display random diffusion or directional displacement and then, are confined at a specific location. In this scenario, it is believed that virus particles first bind to their surface attachment factors and/or primary receptor and then actively move to a plasma membrane microdomain to interact with a secondary receptor to be internalized. This type of movement has been described for both influenza A virus (IAV) and mouse Polyomavirus (mPy). In the case of IAV, the virus particles first land on the cell surface and move to their final internalization site in an actin-dependent manner. After arrest, the virus particles enter the cells via CME [29]. Study of mPy entry revealed that immediately after binding to the cell surface, the virus displays a free diffusion mobility profile that is rapidly replaced by a combination of confinement and drifting [30]. Inhibition of the free diffusion by cholesterol depletion inhibited viral infection, suggesting that the diffusion step is crucial. It has been proposed that during the diffusion step, mPy binds multiple copies of its ganglioside receptor and maybe other receptors (Figure 2B). This increased affinity between the virus particle and its receptor(s) prevents free diffusion of the virus and confines the virus particle to a specific microdomain on the plasma membrane. This confinement is dependent on cortical actin [30]. After confinement, the viral particle will be able to be endocytosed. It is important to highlight here, that ganglioside clustering was not monitored during mPy entry. Therefore the clustering model was proposed in analogy to the Cholera toxin-induced clustering of ganglioside [31]. Similar mechanisms of confined mobility have been described for the simian polyomavirus 40 (SV40) after binding to the ganglioside GM1 [32].

A more recent study has focused on the internalization of canine parvovirus (CPV), which takes place in a clathrin-dependent manner [18]. In this work, the authors were able to reveal that CPV, after cell surface attachment, actively diffuses at the surface of the cell and within 30s interacts with an already existing coated pit (Figure 2C). As such, these viruses use a very unique strategy by hijacking pre-formed endocytic structures. This type of hijacking has been previously shown to be used by dengue virus (DV) [33], however the CPV entry study is one of the rare works where the authors were able to correlate the number of receptors attached to a virus particle to the diffusion rate and endocytic rate of these particles. Remarkably, the authors were able to estimate that individual CPVs were bound to fewer than three Transferrin (Trf) receptors per viral particle and that interaction with the Trf receptor took place prior to falling into clathrin-coated pits. Interestingly, virus particles that were bound to more than eight receptor molecules were not able to diffuse and failed to enter cells by CME. The affinity of CPV for Trf receptor was shown to be much lower than the affinity of Trf itself. This lower affinity might allow CPV to limit the number of bound Trf receptor. As such, although viruses are believed to bind more than one receptor per particle due to their polyhedral nature, there might be an optimum stoichiometry between the receptor and the virus particle. On one hand, virus/receptor binding is crucial for diffusion and endocytosis, but on the other hand, binding too many receptors to a single virus particle might severely affect the diffusion rate of the virus/receptor complex and be detrimental for virus entry. In the case of CPV bound to the Trf receptor and SV40 bound to GM1, the diffusion rate of the virus/receptor complex is about 10 times lower than the receptor/natural ligand complex. Whether this decreased diffusion rate promotes better or less efficient endocytosis of the virus/receptor complex compared to the natural ligand/receptor complex and whether this is favorable or detrimental to virus infection remains to be shown. We can speculate that, for viruses whose entry relies on the formation of *de novo* endocytic structures, binding multiple copies of their receptors would be beneficial (e.g., reovirus, VSV, IAV). This binding would then limit their diffusion rate on the plasma membrane giving the cell time to form an endocytic structures at their vicinity. Limiting the number of bound receptors will allow the virus particles to maintain a greater diffusion rate thus increasing the probability of hijacking a pre-existing endocytic structure (like CPV).

## 5. Redistribution of Virus Particles to Permissive Endocytic Sites

Virus mobility at the surface of the host cell membrane is usually linked to intrinsic mobility of the proteins and/or lipid receptors to which they bind. As mentioned above, viruses have found ways to alter their diffusion rate by binding multiple copies of the receptors that in turn result in a decreased diffusion rate. In some cases, viruses use more complex strategies to control their mobility at the cell surface. Binding to a receptor can lead to the activation of signaling pathways leading to redistribution of the virus/receptor complex. This is often dependent on the underlying cytoskeleton.

Many viruses have been described to land on the cell surface and actively relocate to different sub-membranous locations where they can be efficiently endocytosed. It has been known for a long time that viruses can associate with membrane cellular projections such as filopodia, microvilli, and lamellipodia [34,35]. With the development of fluorescent live microscopy, researchers have been able to demonstrate that particles of the Murine Leukemia Virus, pseudotyped with either the Avian Leukosis Virus (ALV) or VSV glycoproteins, are actively transported on the surface of filopodia in an actin- and myosin-dependent manner toward the cell body. Only after being localized to the cell body, at the base of the filopodia, viruses are able to enter the cell [19]. Similarly, infection of cells by HPV-16 was shown to be partially dependent on the active transportation of the virus particles at the surface of filopodia toward the cell body [36].

Recent work has shown that many bunyaviruses make use of the C type lectin DC-SIGN as an endocytic receptor to target and enter dendritic cells [9,37]. The DC-SIGN protein is localized at the very leading edge of dendritic cells where it clusters. Interestingly, endocytosis of DC-SIGN takes place at sites posterior to the leading edge. Tracking of labeled DC-SIGN revealed the active linear redistribution of the receptor from the leading edge to the lamellar posterior endocytic sites [38]. Using the bunyavirus Uukuniemi virus (UUKV), it was possible to visualize the virus/DC-SIGN interactions in live cells and analyze their dynamics, which showed that additional lectin molecules were recruited to the virus binding site [9].

A good example of virus-induced virus/receptor complex relocation is during the entry of group B coxsackieviruses (CBVs) in epithelium cells. During the natural course of infection by CBVs from the fecal-oral route, CBV binds its co-receptor DAF located at the apical membrane of epithelium cells. This initial binding induces the tyrosine kinase Abl that in turn induces the Rac-dependent reorganization of the actin cytoskeleton. This process allows the dynamic redistribution of the virus/DAF complex to the tight junctions where the virus can interact with its receptor, coxsackievirus and adenovirus receptor (CAR) (Figure 2D). Binding of viruses to CAR induces conformational changes in the viral capsid that are fundamental for CBV entry [39].

Similarly human adenovirus engages its receptor CAR and its co-receptor αV integrin at the apical membrane of polarized cells. The interaction of adenovirus with CAR is associated with diffusion and actin-myosin-2-dependent drift whereas association of adenovirus with integrins induces the local confinement of adenovirus at the surface of the cell. Single virus particle tracking revealed that first adenoviruses diffuse at the surface of the cells by primary interaction with the CAR receptor. After a few minutes, the virus particle diffusion rate dramatically decreases as drifting and confined motion increases suggesting that the virus interacted with the co-receptor αV integrin [40]. In this case, the authors propose that the tug-of-war between integrin-associated confinement and the CAR-associated diffusion favors a physical shedding of the adenovirus fiber proteins. The loss of these fibers will allow for exposure of internal proteins, which are fundamental for membrane rupture and endosomal escape [41]. This finding opens up a novel dimension of virus mobility at the cell surface. Confined or directional motion of viruses is not solely for viruses to interact with receptors or to move to permissive sites for internalization but can be directly linked to virus maturation.

## 6. Signaling in the Host Cell during Viral Entry

It is evident that signaling is an important part of the very first steps of virus/receptor interactions and therefore virus entry. A simple explanation would be that these signaling pathways are activated upon virus binding to receptor(s) and would in turn actively induce internalization of the virus/receptor complex. This process would be referred as receptor-mediated signaling-induced endocytosis. Although this notion of induced endocytosis is largely accepted, very little evidence exists that correlates induction of signaling (*i.e.*, phosphorylation of receptor tyrosine kinases) and physical uptake.

Viral infections are typically associated with major impacts in the molecular physiology of host cells, often disturbing the expression of cellular genes and leading to an increased level of stress proteins and the activation of the innate immune system. These profound changes result, with no doubt, from the perturbation of the cell signaling networks induced by the infection itself. In many cases, viruses also usurp actively the signaling systems of host cells to create a favorable environment for their own replication and amplification [5,16]. A number of evidence supports the view that signaling begins at the cell surface following binding of viruses to receptors. Depending on the virus, receptors, and host cells, initial attachment can lead to activation of serine, threonine, tyrosine, and other kinase pathways. As a consequence, cascades of downstream responses ensue in the cytoplasm, and eventually, also in the nucleus. Signaling induced by viruses then relies on the usual cellular second messengers (phosphatidylinositides (PIs), diacylglycerides, and calcium) and on various regulators of membrane intracellular trafficking and actin cytoskeleton dynamics.

Studies on the internalization of IAV and VSV have revealed that these viruses endocytose via CME. As opposed to DV and CPV that hijack pre-existing clathrin endocytic structures, IAV and VSV are internalized by formation of *de novo* clathrin structures under individual virus particles [25,29]. Interestingly, the rate of formation of these endocytic clathrin structures was found to be between 16–20 times higher at the sites of membrane bound viruses compared to clathrin structures lacking virus particles [25,29]. The molecular mechanisms leading to this local increase of internalization rate remain to be determined.

SV40 is an excellent example to illustrate the complexity of virus-induced signaling in viral entry. Following attachment to major histocompatibility complex class I and GM1, virus particles trigger local activation of tyrosine kinases [42,43,44]. As a consequence, actin filaments are reorganized and the particles are internalized in caveolin-1-positive or lipid-raft vesicles [44,45,46,47]. More than 50 different kinases have been identified to regulate the entry of SV40 and early steps of its infection [48].

Adenoviruses 2 and 5 (Ad2 and Ad5) have also been characterized as relying on signaling for entry. Both viruses use CAR and the integrin αvβ3 as receptors to undergo internalization and both rely on endosomal acidification for productive infection [5,49]. The interaction with αvβ3 integrin activates the PI3 kinase (PI3K) that in turn induces the synthesis of PI(3,4)P2 and PI(3,4,5)P3. These lipids are subsequently responsible for the activation of protein kinase C (PKC) and small GTPases such Rabs and Rho. PKC and the GTPases then promote actin polymerization, rapid increase in fluid internalization by macropinocytosis, and clathrin-dependent uptake of adenovirus particles [50,51]. The findings that activation of these GTPases would induce CME of the virus particles have to be carefully addressed since recent studies demonstrate that CME is actin independent [52,53].

Echovirus 1 (EV1) is also known to use integrins for its internalization. EV1 binds to the collagen receptor α2β1. It has been shown that binding of collagen to α2β1 causes conformational changes within the integrin, which leads to p38 activation. Interestingly, EV1 prefers the inactive conformation of α2β1 for its binding and does not lead to p38 pathway activation suggesting that the virus will use the receptor differently than the natural ligand [54].

Several viruses have been proposed to subvert the signaling and endocytic activities of the epidermal growth factor receptor (EGFR) for their cell entry [55]. EGFR is a highly dynamic receptor tyrosine kinase that continuously travels back and forth between the plasma membrane and endocytic vesicles. EGFR-mediated signaling is believed to promote the penetration of some herpesviruses such as the human cytomegalovirus (CMV) and herpes simplex virus 1 (HSV1) [56,57]. Following attachment to ανβ3 integrin, interactions of the CMV glycoprotein gB with EGFR triggers the PI3K pathway [56]. In coordination with the activation of Src signaling, through the virus binding to ανβ3 integrin, RhoA and cofilin are activated. This series of events results in a local F-actin rearrangement that is believed to facilitate the passage of the virus through the actin cortex. HSV1 follows a similar strategy [57]. Another herpesvirus, Epstein-Barr virus (EBV), may also subvert the EGFR biological functions. EBV has been shown to induce actin reorganization through activation of PI3Ks [58,59]. However, the demonstration of the EGFR involvement in this process is missing.

**Figure 3:**
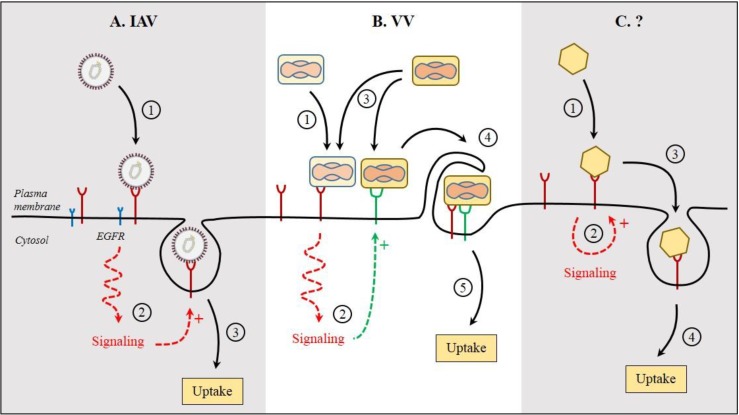
Signaling during virus entry. Infections cause profound perturbations of the host cell signaling networks. Signaling likely begins at the plasma membrane, after virus binding to receptors. Here are few examples of signaling during virus entry. (**A**) Following attachment at the cell surface, influenza A virus (IAV) relies on the activation of epidermal growth factor receptor (EGFR) for the endocytosis of virions. However, no direct interaction has been reported between IAV and EGFR; (**B**) In the case of vaccinia virus (VV), virions seem to cooperate for entry. In this model, the first incoming virions trigger signaling through binding to primary receptor(s). The signal facilitates the recruitment of other receptors, which all together mediate the attachment and endocytosis of further incoming particles by induction of micropinocytosis; (**C**) Upon binding of viruses to receptor molecules, receptor-mediated signaling induces the local internalization of the virus/receptor complex. Concrete evidence supporting such receptor-mediated signaling that results in an active internalization is missing. The encircled numbers indicate the sequence of events.

A more direct role in the entry and internalization of IAV and hepatitis C virus (HCV) has been attributed to EGFR. Following attachment of IAV to the cells, the activation of EGFR and downstream signaling via PI3Ks have been proposed to be required for the endocytosis of the virions (Figure 3A) [60]. The binding of HCV to one of its receptors, CD81, is believed to allow for interactions with EGFR, and subsequently, for internalization of the virus/EGFR complex [61,62]. The activation of EGFR signaling seems to enhance the entry of further HCV particles. In addition to these viruses, human papillomavirus type 16, respiratory syncytial virus, and African swine fever virus have the ability to hijack the EGFR functions to ensure their entry [63,64,65,66]. Many of these viruses share a dependence on membrane blebbing and rapid rearrangement of actin filaments for infectious entry.

The Kaposi’s sarcoma-associated herpesvirus (KSHV) is another excellent example of a virus that relies on signaling and actin reorganization for entry [67]. The ectodomain of the surface envelop glycoprotein gB of KSHV contains a Arg-Gly-Asp motif (RGD), which is recognized and used by the integrin α3β1 for virus binding and entry [68,69,70]. Interaction between gB and the integrin activates focal adhesion kinase (FAK) and Src kinases leading to PI3K and Rho GTPase activation [71,72,73,74,75,76]. In addition, KSHV induces the extracellular signal-regulated kinase 1 and 2 (ERK1/2) through the PI3K-PKCζ-mitogen activated or extracellular regulated kinase (MEK) pathways [72,77,78]. The activation of these pathways results in a dramatic rearrangement of the actin cytoskeleton, which leads to the internalization of virus particles by macropinocytosis into human fibroblasts [79,80].

Vaccinia virus likely represents the best evidence for a direct role of virus-induced signaling in uptake of virions through macropinocytosis. After reaching the plasma membrane, vaccinia virus activates a complex signaling network that involves PAK1 and leads to major alterations in the actin cortex and membrane blebbing, which result in the internalization of particles by macropinocytosis [81]. The virus has developed a strategy based on mimicry of apoptotic bodies to trigger the signaling, blebbing, and macropinocytosis to enter cells. Phosphatidylserine in the viral membrane is essential to induce these cellular mechanisms. Interestingly, vaccinia virions seem to cooperate during the early stages of entry (Figure 3B) [82]. The first incoming virions may act as a decoy to induce the signalization that will facilitate the entry of further particles through macropinocytic engulfment [83]. This underlines the importance to investigate virus infection experiments at the single cell level and to understand how timing of signal-induced response affects host cell and virus entry.

It is clear that virus binding and internalization represent a series of events that involves hundreds of cellular factors, which are highly dynamic, interconnected, and coordinated in time and space. A predominant part of the information available is for viruses that depend, directly or indirectly, on macropinocytosis for entry but, it is not always clear whether macropinocytosis is used only as an entry pathway or rather as a means to disrupt the actin cortex to facilitate virus entry through other endocytic routes. Further studies are required for viruses, which use alternative pathways for infection. For most viruses, many basic but important questions remain open (Figure 3C). (1) Does the activation of these signaling pathways promote the internalization; (2) Is the activation a consequence of the virus internalization; (3) Does the virus-induced receptor-dependent signaling trigger a cascade of events that leads to the internalization of both the virus and its receptor; (4) or is the internalization signal-independent; (5) How is the signal passed from the receptor ectodomain to the cytosolic tail following virus binding; (6) What are the signal sequences in the cytoplasmic tail of the receptors; and (7) What are the cellular factors required for endocytosis?

## 7. Endocytic Signals and Signaling Motifs in Virus Receptors

In the past decades, comparisons of the cytoplasmic tail of many receptors that use clathrin for endocytosis support the view of a high diversity of endocytic motifs rather the existence of common endocytic motifs [84]. The motifs in the cytoplasmic tail of receptors can be either linear, conformational, and/or covalently modified. The linear motifs are composed by variant and invariant amino acids. Examples are the motifs YXXΦ and [FY]XNPX[YF] as well as motifs composed of dileucine (LL) and acidic clusters, where the single-letter code indicates amino acids, X any amino acid, F an amino acid with a bulky hydrophobic side chain, and brackets either amino acid is allowed at this position [85,86,87]. While the invariant amino acids are the most critical, the function of these signals can be influenced by the variant amino acids, the flanking sequences, the phosphorylation of residues in or near the motif (S, T, or Y), the spacing from the transmembrane domain, *etc.* [88,89,90,91]. Other motifs can be conformational determinants under the form of amino acid patches in folded domains [92]. In contrast to linear signals, conformational motifs appear to be unique for a specific cargo such as those described in VAMP-7 and R-SNAREs [92,93]. Lastly, covalent modifications such as phosphorylation (S, T, or Y) or polyubiquitination (K) can also serve as endocytic signals [94,95]. However, the endocytic motifs and other signaling sequences remain largely unknown for most clathrin-independent pathways. In many cases, it is not clear whether endocytic signals are simply docking sites for adaptor proteins or rather participate to cell signaling.

Regarding virus entry, little information is available about the endocytic and signaling motifs important for the uptake and sorting of virus particles into endocytic compartments. Experimental evidence for a direct role of such motifs in receptor-mediated internalization of virions is rare. In this respect, DC-SIGN represents one of the most interesting and documented virus receptor models. This cell surface type II transmembrane protein can bind to multiple viruses from various viral families through high-mannose *N*-glycans [96]. Though relatively short, the cytoplasmic tail of the lectin carries several motifs involved in signaling, endocytic internalization, and intracellular trafficking such as S, T, and Y residues, but also, an acidic cluster (EEE) as well as YXXΦ and LL-based motifs [96]. Interestingly, recent studies have shown that DC-SIGN is able to trigger selective signal transduction pathways, which seem to depend on the nature and glycosylation pattern of the captured antigens [97,98]. It is tempting to postulate that viruses may usurp such biological functions of a receptor to trigger diverse cell signaling cascades for their own sorting into a specific endocytic pathway.

**Figure 4:**
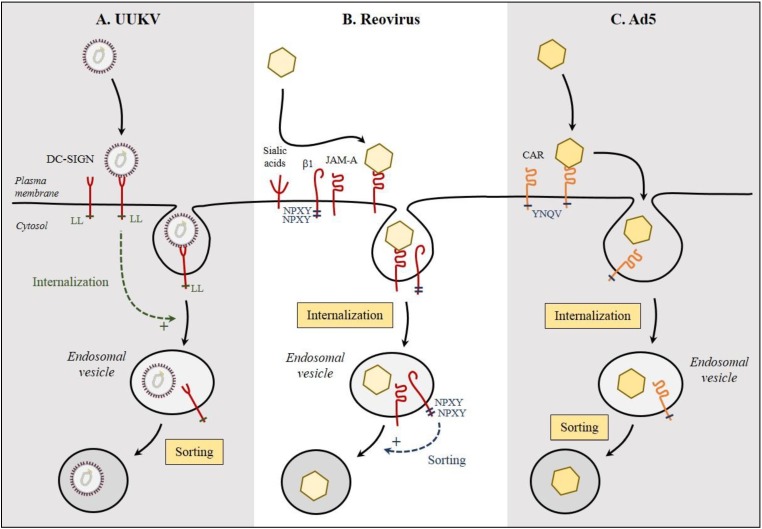
Endocytic signals in virus receptors. Relatively few endocytic signals in receptors have been assessed for their role in virus entry and infection. (**A**) The LL motif of DC-SIGN is critical for Uukuniemi virus (UUKV) internalization and acts as endocytic motif; (**B**) The two NPXY motifs in the β1-integrin (β1) seem to act as sorting motifs to direct reovirus into the right endosomal compartment; (**C**) No function has been attributed to the YNQV motif in CAR regarding infection by adenovirus 5 (Ad5).

By interacting with specific adaptor proteins, endocytic motifs determine in general the internalization pathways of cargo, and possibly of viruses. CME represents the best-documented sorting processes of cargo from the plasma membrane into the endocytic machinery. As previously mentioned, DC-SIGN contains a LL-based motif in the cytoplasmic tail, which acts as a typical docking site for adaptor proteins required for the formation of clathrin-coated pits, and therefore, receptor-induced CME [84,99]. The LL motif is critical for the endocytic activity of DC-SIGN [9,100]. Recent work has shown that UUKV is no longer able to enter cells expressing the endocytic-defective LL->AA mutant of DC-SIGN (Figure 4A) [9,101]. This is the only evidence of a direct role for DC-SIGN, beyond attachment, in productive virus internalization [96,100,102]. A role of additional cellular transmembrane factors in the endocytosis of virus particles cannot be however completely ruled out, as it seems that measles virus, which also binds DC-SIGN, requires additional interactions with CD150 for infectious entry [103].

To our knowledge, there are only a few reports about endocytic motifs that are important for virus entry. The β1 integrin that serves as co-receptor for reovirus has a cytoplasmic tail with two NPXY motifs, known for their role in CME. Cells that have modified NPXY motifs lead to a reduction in reovirus infectivity [104]. However, neither virus binding nor endocytosis is impaired, suggesting a role of the NPXY motifs as sorting signals into appropriate endocytic vesicles following the internalization of particles (Figure 4B). Similarly, in the case of Ad5, the YNQV motif in the cytoplasmic tail of the virus receptor CAR does not seem to impact the entry of virus particles (Figure 4C) [105]. It is not known whether the alteration of this motif also results in a misrouting of internalized virus particles. In general, the role of sorting motifs are hard to investigate. After decades of research based on inactivation of signaling/endocytic motifs in the EGFR cytoplasmic tail, the exact signals created by the binding of the natural ligands to EGFR remains largely a matter of debate [106].

## 8. Concluding Remarks

In this review, we have summarized current knowledge of the early virus-host cell interactions from virus attachment on the cell surface, including interaction with receptor and induction of signaling, to uptake. While virus/receptor interactions and the subsequent intracellular trafficking of virus particles have been extensively studied, it is apparent that little work has addressed the transition processes between the extracellular and intracellular stages, and a substantial number of issues remains unresolved [16,107].

Receptor-mediated endocytosis of viruses is well established, as internalization of virus particles is fully dependent on the presence of virus-specific surface exposed cellular receptors. Viruses can only infect cells that display their specific receptors. Whether and how virus/receptor interactions induce specific signaling (receptor-mediated signaling) that promotes internalization of the viral particle is much less understood and it remains unclear for many viruses whether the entry-associated signaling is a cause or a consequence of the entry event. Some viruses seem to not induce active signaling, however this does not necessarily imply that these viruses are randomly endocytosed in a completely passive manner. It has been suggested that the multivalent nature of the viral capsid (e.g., the possibility of binding multiple copies of the receptor) could constitute a “signal” that leads to particle internalization. This virus-induced receptor clustering could increase the probability of the virus/receptor complex to be up taken by stabilizing the formation of the endocytic structure [7]. Recently viruses that bind lipid-based receptors, such as mPy and SV40, have been proposed to locally induce lipid clustering, which, in turn, causes local membrane curvature, and thereby, the endocytosis in a lipid raft dependent manner [108,109]. Interestingly, a similar model was proposed for human norovirus upon binding of the glycosphingolipids receptor [110]. The importance of the lipid receptor is crucial in this mechanism but it is possible that other viruses have developed alternative strategies to induce membrane curvature. We can speculate that some viral receptors have affinity for specific lipids or for specific membrane binding proteins that will in consequence induce local membrane curvature.

It is frequently unknown whether viruses induce the same receptor clustering and signaling as the natural ligand of the receptors that they hijack. The reason is probably due to the absence of information about the signals produced by natural ligands themselves. EV1 is thus far the sole documented example of a virus that uses an endocytic pathway distinct from that of the natural ccligand [54]. Similarly, it is often not known whether the receptor clustering and the subsequent endocytic routes are cargo dependent. Recent examples suggest that the endocytic pathway is virus specific. While both Ad2 and Ad5 use CAR and αV integrin as receptors, Ad2 seems to enter cells by macropinocytosis and Ad5 through CME [40,50].

Cell surface receptors, such as Trf receptor, GM1, and other gangliosides, often lack known signaling/endocytic motifs or a prominent cytoplasmic tail. In the latter case, this strongly suggests the involvement of additional co-receptors or microdomains in which endocytic receptors would mediate the internalization of virus/receptor complexes. The adaptor and coat proteins associated with such processes are also poorly characterized for most endocytic routes and viruses. The use of different adaptors likely generates different downstream signals, which can be useful for viruses, both in the short term, by triggering uptake, and in the mid-term, by creating a favorable and beneficial environment for the establishment of infection.

While much work has focused on signaling that emanates from the plasma membrane, it is not clear whether virus-dependent signaling can occur through internalized receptors. An increasing number of viruses have been reported to remain attached to their receptors after uptake or become attached to their receptors within endosomal vesicles, at least during the early steps of the endosomal maturation [3]. Recent studies of bacteria suggest that the location of ligand/receptor interactions dictates the type of signals produced. The signaling induced by the bacterial coat protein LPS through TLR4 involves different adaptors, the identity of which depends on whether interaction occurs at the plasma membrane or within endosomes [111,112]. This leads to the possibility that viruses can also signal at multiple locations during their entry.

Many questions about virus entry remain open (1) Whether viruses use endogenous endocytic pathways or are rather able to subvert the cell machinery to promote their own, specific entry route; (2) Whether virus uptake occurs via a constitutive endocytic process or through a ligand-induced endocytic mechanism; and (3) What are the benefits for viruses to use one of these possibilities? It clearly appears that the results gleaned from virus studies may provide valuable information, not only about viral infections, but also about hard-to-investigate, basic mechanisms that control the first stages of cargo uptake and signaling into the cells.
